# 

**Published:** 1862-03

**Authors:** 


### POPULAR ARTICLES.

In the February number, in the first editorial, we gave a few plain directions in regard to the method of using artificial teeth. We intended these directions for those whom they more immediately concern, viz.: the wearers of artificial teeth, rather than for the profession.

Every operator will fully understand the value of some plain, definite, understandable directions, in such a form that he could give to his patients, that they might study and consider at their leisure, and that would be far more valuable to them than a few hasty verbal directions.

In the present number we give an article of the same character, and for the same object, on the "Care of the Deciduous Teeth." We issue extra sheets of these articles, to put into the hands of our patients, as opportunity may afford.

We propose in the next number to present, with the same object, the subject of insuring dental operations.

If any of our brethren should wish any copies of these articles for distribution to their patients, we will with pleasure furnish them at the cost of publication, and postage. T.

### THE REGISTER—BACK NUMBERS.

We have on hand one complete set of the Dental Register, neatly bound, which we will dispose of at $3.00 per volume for the first eight volumes, and $3.50 per volume for the remaining seven.

We have also a number of copies of vol. 15, neatly bound, at $3.50 each, which would be better for those ordering that volume, than to get it in numbers. We will send by mail or express, as may be desired. T.

### STILL AHEAD.

Who? Why, S. S. White, of course. He always was ahead of other people; but this time he has got ahead of himself, or at least ahead of many of his former achievements. If you don't believe it, reader, try his new "vulcanite" teeth with the patent rivets. That's the way we found it out. There oughtn't to be any *artificial* thing better than they are. W.

### REFERENCE.

Frequent inquiries have been made recently in regard to the prosecution of J. T. Toland by the American Hard Rubber Co. for infringement of its patents.

We would refer all such inquirers to the February number of the Register. We there endeavored to give a correct statement of the present position of the case. T.

May.5.1862

### CONTENTS.

### DENTAL REGISTER,

### OF THE WEST.

**Issued Monthly, at $3 a year in advance.**

DEVOTED TO THE INTERESTS OF THE DENTAL PROFESSION.

**Edited by J. TAFT and GEO. WATT.**

The Volume commences in January and closes in December.

The Register being issued monthly is always fresh, therefore indispensable to the practitioner who desires to keep posted up with the new inventions and improvements which are daily developed. Neither expense nor effort will be spared to give value and variety to its contents. Articles will be illustrated with well executed engravings, whenever they will add to the interest or assist in explanation.

Its extensive circulation and frequency of issue makes it the BEST DENTAL ADVERTISING MEDIUM in the United States.

☞CONTRIBUTIONS SOLICITED.

Address Original Communications to **Geo. Watt, Xenia, O.**

Exchanges to **J. Taft**, or **Dental Register, Cincinnati, Ohio.**

Business Communications to

J. TAFT, Publisher,
56 West Fourth Street, Cincinnati, O.

JNO. T. TOLAND, Proprietor,
38 West Fourth Street, Cinninnati, O.

Commmunications must reach the Editor as early as the 15th, to insure insertion in the next issue.

